# Assessment of lactic acid bacteria isolated from the chicken digestive tract for potential use as poultry probiotics

**DOI:** 10.5713/ab.22.0455

**Published:** 2023-05-02

**Authors:** Merisa Sirisopapong, Takeshi Shimosato, Supattra Okrathok, Sutisa Khempaka

**Affiliations:** 1 School of Animal Technology and Innovation, Institute of Agricultural Technology, Suranaree University of Technology, Nakhon Ratchasima 30000, Thailand; 2 Department of Biomolecular Innovation, Institute for Biomedical Sciences, Shinshu University, Kamiina, Nagano 399-4598, Japan

**Keywords:** Chicken, Isolation, Lactic Acid Bacteria, *Lactobacillus*, Probiotic

## Abstract

**Objective:**

The use of probiotics as an alternative to antibiotics in animal feed has received considerable attention in recent decades. Lactic acid bacteria (LAB) have remarkable functional properties promoting host health and are major microorganisms for probiotic purposes. The aim of this study was to characterize LAB strains of the chicken digestive tract and to determine their functional properties for further use as potential probiotics in poultry.

**Methods:**

A total of 2,000 colonies were isolated from the ileum and cecal contents of the chickens based on their phenotypic profiles and followed by a preliminary detection for acid and bile tolerance. The selected 200 LAB isolates with exhibited well-tolerance in acid and bile conditions were then identified by sequencing the 16S rDNA gene, followed by acid and bile tolerance, antimicrobial activity, adhesion to epithelial cells and additional characteristics on the removal of cholesterol. Then, the two probiotic strains (*L. ingluviei* and *L. salivarious*) which showed the greatest advantage *in vitro* testing were selected to assess their efficacy in broiler chickens.

**Results:**

It was found that 200 LAB isolates that complied with all measurement criteria belonged to five strains, including *L. acidophilus* (63 colonies), *L. ingluviei* (2 colonies), *L. reuteri* (58 colonies), *L. salivarius* (72 colonies), and *L. saerimneri* (5 colonies). We found that the *L. ingluviei* and *L. salivarius* can increase the population of LAB and *Bifidobacterium* spp. while reducing *Enterobacteria* spp. and *Escherichia coli* in the cecal content of chickens. Additionally, increased concentrations of valeric acid and short chain fatty acids were also observed.

**Conclusion:**

This study indicates that all five *Lactobacillus* strains isolated from gut contents of chickens are safe and possess probiotic properties, especially *L. ingluviei* and *L. salivarius*. Future studies should evaluate the potential for growth improvement in broilers.

## INTRODUCTION

The poultry industry is one of the fastest growing sectors of global livestock production. Various aspects (such as breed, nutrition, animal health, etc.) are used to develop all segment chains to improve potential production efficiencies [1]. However, due to the high efficiency of meat or egg production, inputs for specific nutrients and health management require more attention. Among the aspects that should be taken into consideration for optimal poultry performance, overall health and proper functioning of the avian gastrointestinal tract (GIT) are crucial [2]. In addition, the intensive poultry production system has led to an increase in stress, which can lead to a decrease in immune function and allow colonization by pathogens [3]. This may pose a serious health hazard to birds and consumers of poultry products as outbreaks of different diseases have resulted in huge economic losses. Therefore, finding alternative feed additives that can effectively control pathogens and retain growth promoting properties would help address these issues. Probiotics are defined as "living microorganisms that, when taken in sufficient quantities, provide a host with health benefits", which play a key role in the development of immunity against pathogens as well as the health and growth of broilers [4], resulting in safe and cost-effective production [5].

Lactic acid bacteria (LAB) are the main source of probiotics used in animal feeds, which have several benefits for the host health including gut microbiota modulation, immunomodulation, anti-inflammatory and antimicrobial effects [6]. LAB have been reported to possess a broad spectrum of beneficial and health promoting properties which influence the intestinal microbial balance of the host to contribute to the regulation of innate intestinal immunity and homeostasis [7]. LAB also produces metabolites such as lactic acid, antioxidants and antimicrobial compounds, especially bacteriocins and short chain fatty acid (SCFA) that contribute to the inhibition of the growth of pathogenic bacteria [8]. LAB including species *Enterococcus*, *Lactobacillus*, *Pediococcus*, *Streptococcus*, *Lactococcus*, *Vagococcus*, *Leuconostoc*, *Oenococcus*, *Weissella*, *Carnobacterium*, and *Tetragenococcus* are natural microflora in the GIT of humans and animals [5] characterized by the production of lactic acid. The main candidate strain introduced for probiotic purposes belongs to the genus *Lactobacillus* which is a major genus of LAB and accommodates more than 200 species [9]. In poultry, feeding *Lactobacillus* probiotic strains improves not only the digestion of feed, but also the absorption of nutrients [3]. In addition, probiotics increase the growth performance, neutralizing various enterotoxins and enhancing the immune responses of poultry [10]. Additionally, probiotics reduce the risk of gastrointestinal colonization by foodborne pathogens, such as *Escherichia coli* (*E. coli*), Campylobacter, *Clostridium*, and *Salmonella* [11] and increase the safety of poultry-based foods due to their diverse advantages, LAB has been chosen as the best candidate for probiotics.

However, not all LAB are probiotics and their characteristics and safety profile also need to be assessed. In order to qualify as probiotics, candidate bacterial strains must be able to tolerate acid and bile, coaggregation with pathogens, antimicrobial activity, adherence to intestinal mucosa, antibiotic resistance, and modulation of intestinal barrier functions [12]. Probiotic strains of the same ecological origin can be more compatible with animal gut microbes, which makes it possible to optimize productive performance [13]. For this reason, native and species-specific probiotics should be considered, in which LAB with health promoting properties are mostly the major components of the chicken intestinal microflora [14]. Therefore, this study aims to isolate and evaluate LAB from the GIT of chickens for future use as highly stable probiotics in poultry diets. The findings of this study would also provide valuable sources of highly efficacy and appropriate probiotics for the poultry industry.

## MATERIALS AND METHODS

All experiments were conducted according to the principles and guidelines approved by the Animal Care and Use Committee of Suranaree University of Technology, Nakhon Ratchasima, Thailand (SUT; Approval number: 042016).

### Sample collection

Bacteria were isolated from the digestive tract of healthy broilers, slow-growing chickens (Korat chickens) and laying hens raised on the farm at Suranaree University of Technology. All birds were fed a standard diet without any antibiotic supplement. After the birds were euthanized by chloroform inhalation, the blood was removed and the peritoneal cavity opened. The digesta contents of the ileum and caeca were separately removed under sterile conditions and transported to the laboratory immediately on ice for microbial analysis.

### Isolation of lactic acid bacteria

Ten-fold serial dilutions of each sample (ileum and cecal digesta) were made by suspension in a phosphate-buffered saline (PBS, pH 7.4). For each dilution, 100 μL was spread plated on De Man, Rogosa and Sharpe (MRS) agar (Oxoid, Basingstok, Hampshire, England) plates. The inoculated plates were incubated under anaerobic conditions using a gas pack at 37°C for 48 h. Isolated pure cultures were evaluated by the catalase test, Gram stain and bacterial morphology. The isolated bacteria with the characteristics of *Lactobacillus* specifications, such as creamy white colony, catalase negative reaction and Gram’s positive rod shape were stored in MRS broth containing 20% (v/v) sterile glycerol and stored at −80°C for further analysis.

#### 16S rRNA gene sequencing for identification

The bacterial isolates that passed the confirmatory tests for *Lactobacillus* were subsequently selected for molecular identification. The *Lactobacillus* strains were grown overnight and the genomic DNA was extracted from the culture using a bacterial genome extraction kit (KOD FX Neo; Toyobo Inc., Osaka, Japan). The 16S rRNA gene was amplified using universal primers as follows: 27F (5′-AGAGTTTGATCCTGGCTCAG-3′) and U1492R (5′-GGTTACCTTGTTACGACTT-3′). The polymerase chain reaction (PCR) amplification had initial DNA denaturation at 94°C for 5 min, followed by 35 denaturing cycles at 94°C for 1 min, annealing at 55°C for 1 min, elongating at 72°C for 1 min, and followed by a final extension at 72°C for 5 min. The 5 μL PCR product was analyzed by electrophoresis in 1% agarose gel at 90 volts for 45 min, followed by staining with a 1% solution of ethidium bromide (50 μL/L) and distaining with Tris-acetate-ethylenediaminetetraacetic acid 1x for 10 min. Gel was visualized by UV transillumination and recorded by digital camera. The sequencing of 16S rRNA gene was conducted using a genetic analyzer. The 16S rRNA gene sequences of strains were automatically compared using BLAST against the sequences of bacteria available in databanks ( http://www.ncbi.nlm.nih.gov/). The phylogenetic analysis was conducted using the neighbor-joining algorithm. After the *Lactobacillus* strains were identified by 16S rRNA sequencing, all strains were then screened for the probiotic properties mentioned below.

### Screening of probiotic properties

#### Tolerance to acidic pH

The resistance of the examined strains under acid conditions was tested as previously described by Heravi et al [8]. Isolated bacteria were grown in MRS broth at 37°C for 18 h, then a subculture was added to fresh MRS broth and incubated for another 24 h. The cultures were centrifuged at 4,000×*g* for 5 min, the pellets washed twice in sterile PBS, pH 7.4, and resuspended in PBS. Each strain was diluted 1/100 in PBS at pH 2.0, 2.5, 3.0, and 3.5 then incubated for 4 h. The bacteria were then transferred to MRS agar incubated anaerobically at 37°C overnight and survival cell counts were determined by plating on MRS.

#### Bile tolerance

Bile tolerance was studied according to the method of Walker and Gilliland [15]. Briefly, isolated bacteria were grown in MRS broth at 37°C for 18 h, then the subculture was transferred into fresh MRS broth and incubated for another 24 h. MRS broth containing 0.3% and 1.0% of oxgall (Sigma-Aldrich, St. Louis, MO, USA) was inoculated with each strain, and incubated at 37°C. The control comprised MRS broth without bile salt. Bacterial growth was monitored by measuring absorbance with a spectrophotometer (Multiskan GO, Thermo Scientific, Vantaa, Finland) at 600 nm at hourly intervals for 6 h. All tests were carried out in triplicate.

#### Antimicrobial activity

A standard agar-well diffusion assay [16] was used to evaluate antagonistic activities against five common chicken pathogens including *E. coli* (ATCC 43888), *Staphylococcus aureus* (ATCC 29213), *Campylobacter jejuni* (ATCC 33291), *Clostridium perfingens* (ATCC 3624), and *Samonella enteritidis* (ATCC 13076) obtained from the American Type Culture Collection (ATCC; Manassas, VA, USA). An overnight culture of each tested pathogen strain was inoculated (0.1%) in Brain-Heart Infusion agar (BHI; Conda-Pronadisa, Madrid, Spain), incubated at 37°C for 16 h. Each of the examined *Lactobacillus* strains were also cultures in MRS broth incubated at 37°C for 18 h as previously described, then harvested by centrifugation (4,000×*g*, 10 min, 4°C). The first supernatant portion (cell-free supernatant) of each isolated strain was neutralized to a pH of 6.5 and the remaining portion was not adjusted for pH, and thereafter both portions were filtered with 0.22 μm membrane filter sterilization. Then normal cells and cell-free supernatant (adjusted or unadjusted pH) of each strain (100 μL) were pipetted into the agar holes (7 mm). The plates were then incubated at 37°C and examined after overnight incubation. Antimicrobial activity was recorded as growth free inhibition zones (mm) around the well. All tests were done in triplicate.

#### Testing antibiotic susceptibility

The testing of antibiotic susceptibility was conducted by isolating all the LAB using the Kirby-Bauer disk diffusion test. Isolates strains were grown in MRS broth at 37°C for 18 h to obtain a density of 10^8^ colony-forming unit (CFU)/mL, then the culture suspension was plated on MRS agar. Antibiotic discs were placed aseptically on the inoculated plates and the agar plates were incubated at 37°C for 24 h. The diameters of the inhibition zones around the discs were measured (in triplicate) and the results were interpreted according to CLSI standard as sensitive (S), intermediate (I), and resistant (R). The antibiotics tested included ampicillin (30 μg), tetracycline (30 μg), chloramphenicol (30 μg) and erythromycin (15 μg) (Oxoid, England).

#### Cell adherence assay

An adhesion assay was conducted as previously reported [17] by using Caco-2 cells. The Caco-2 cells were grown in Dulbecco’s modified eagle medium (DMEM; Gibco, Grand Island, NY, USA) supplemented with 10% (v/v) heat-inactivated fetal bovine serum, penicillin (100 U/mL) and streptomycin (0.1 g/L; Gibco, USA) at 37°C in 5% CO_2_. The culture medium was replaced every 48 h to maintain single-layered Caco-2 cells in the culture plates. Six well tissues were washed twice with PBS and 2 mL of DMEM was added to each well. Plates were incubated at 37°C for 1 h. After incubation, DMEM was removed from each well and replaced with 1 mL of bacterial suspension. After 90 min incubation at 37°C, the wells were washed 3 times with PBS to remove non-adherent bacteria. The washed monolayer was treated with 1 mL of 0.05% aqueous solution of Triton X-100 for 10 min for the lysis of the cells. The number of viable attached bacteria was determined by plating a 10-fold series dilution of a mixture consisting of denatured Caco-2 cells and bacterial cells on MRS agar after 48 h of incubation at 37°C. The adhesion capacity of the species on Caco-2 cells was calculated as the percentage of viable bacteria based on the initial population.

#### Cholesterol removal ability

Isolated *Lactobacillus* was used to remove cholesterol as previously reported by Liong and Shah [18]. Briefly, each isolated strain at 1.0% was inoculated into a freshly prepared MRS broth containing 0.3% oxgall and incubated at 37°C for 24 h. Subsequently, cells were harvested by centrifugation (5,000×*g*, at 4°C, 20 min) and washed twice with sterile water. To prepare heat-killed cells, cell pellets were suspended in 10 mL of sterile water and autoclaved at 121°C for 15 min. Heat-killed cells were further suspended in MRS broth supplemented with 0.3% oxgall acid and 100 μL/mL of water-soluble cholesterol and incubated for 24 h at 37°C. To prepare resting cells, cell pellets were suspended in 10 mL sterile phosphate buffer (0.05 M, pH 6.8) containing 0.3% oxgall acid and 100 μL/mL of water-soluble cholesterol and incubated for 24 h at 37°C. To prepare growing cells, the fresh MRS broth was supplemented with 0.3% oxgall as bile salt. Water-soluble cholesterol was then filter-sterilized and added to the broth at a final concentration of 100 μg/mL, inoculated with each isolated strain (at 1%) and anaerobically incubated at 37°C for 24 h. Subsequently, the mixtures were centrifuged and the cholesterol concentrations in the supernatants were measured using spectrophotometry. All tests were conducted in replicate.

### Evaluation of probiotics *L. ingluviei* and *L. salivarius* in broiler chickens

After the *in vitro* screening, the two isolated *Lactobacillus* strains (*L. ingluviei* and *L. salivarius*) which were the most stable to the treatments with high possible potential, but still a lack of information, were primarily efficacy studies in broiler chickens.

#### Broiler chicken care and management

Thirty one-day-old broilers were used for the *in vivo* evaluation. The chicks were randomly divided into three groups of ten birds each. The birds were orally gavaged once daily with PBS, *L. ingluviei* or *L. salivarius* from day 1 to 14. The three treatments were: i) control group (gavaged with PBS), ii) gavaged with *L. ingluviei* (1 mL/d, 1×10^8^ CFU/mL), and iii) gavaged with *L. salivarius* (1×10^8^ CFU/mL). On day 14, the chicks were injected intraperitoneally with lipopolysaccharide (LPS) 1 mg/kg chicken. The chicks received continuous light for 23 hours per day for day 1 to 10 days which was reduced to 18 hours per day from day 11 onwards. Birds were vaccinated against Newcastle disease and Infectious Bronchitis on day 7. All chickens in each treatment received the same basal diet without anticoccidial drugs. The diets were formulated to meet or to exceed the minimum nutrient requirements of broiler chickens as recommended by NRC [19] and Cobb broiler management guide [20] for starter (0 to 10 days) and grower (11 to 14 days) periods. Feed (mash form) and water were provided *ad libitum* throughout the experimental period. Nutrient composition of experimental diet is presented in Table 1.

#### Sample collection

After 24 hours of LPS injections (day 15), six chickens were randomly selected from each group, euthanized by exsanguination and their cecal content was immediately collected for further analysis of the cecal microbial population and SCFAs.

#### Cecal microbial population analysis by qRT-PCR

The contents of the cecal digesta were used to quantify *Lactobacillus* spp., *Bifidobacterium* spp., *Enterobacter* and *E. coli*. Bacterial DNA was isolated using the QIAamp Fast DNA Stool kit (Qiagen Inc., Hilden, Germany) following the manufacturer’s instructions. The extracted DNA was quantified with a Nano Vue Plus Nano Drop spectrophotometer (GE Healthcare, Chicago, IL, USA) to assess purity and concentration. The populations of cecal microbes were analyzed by quantitative real-time PCR (qPCR). The extracted DNA was used as DNA templates for PCR amplification. The qPCR assay was performed with a LightCycler 480 Instrument II (GmbH; Roche Diagnostics, Mannheim, Germany). The PCR reaction was performed in a white LightCycler 480 Multiwell Plate 96 plates (Roche, Germany) with a final volume of 10 μL using a LightCycler 480 SYBR Green I Master. Each reaction included 5.0 μL of 2×SYBR Green Master Mix, 0.4 μL of 10 μM forward primer, 0.4 μL of 10 μM reverse primer, 2.0 μL of DNA samples and 2.2 μL of nuclease-free water. Each sample was analyzed with triplicate reactions. The reaction conditions for amplification of DNA were initial denaturation at 94°C for 5 minutes, followed by 40 cycles of denaturation at 94°C for 20 seconds, then primer annealing at 50°C for *E. coli*, 58°C for *Lactobacillus* spp., 60°C for *Bifidobacterium* spp. and *Enterobacter* for 30 seconds respectively, and extended at 72°C for 20 seconds [21]. To confirm the specificity of amplification, a melting curve analysis was carried out after the last cycle of each amplification. Absolute quantification of the cecal microbial population was achieved using standard curves constructed by amplification of the known amount of target bacterial DNA.

#### SCFA analysis

The concentration of SCFA (acetic, propionic, isobutyric, butyric, isovaleric, and valeric acid) was analyzed according to the method of Mookiah et al [22]. The cecal digesta was treated with 24% meta-phosphoric acid in 1.5 M H_2_SO_4_ and vortexed to mix. The samples were left at room temperature overnight, then centrifuged at 10,000×g, at 4°C for 20 min, and the supernatant was used for the next step. The analysis was conducted with a gas chromatograph (Agilent 7890B; Agilent Technologies, Santa Clara, CA, USA), with flame ionization detection and nitrogen as the carrier gas. A fused silica capillary column was also used (0.32 mm ×25 m; CP-Sil 5 CB column; Agilent J&W GC Column, Santa Clara, CA, USA).

### Statistical analysis

Data were analyzed using the one-way analysis of variance of SPSS version 18.0 (SPSS, Inc., 2010). Significant differences among treatments were assessed by Tukey’s post hoc test. A threshold level of p<0.05 was used to determine the significance.

## RESULTS

### Isolation of lactic acid bacteria

A total of 2,000 colonies were isolated from the ileum and cecal contents of the chickens based on the preliminary identification of LAB using the criteria of a creamy white colony and Gram’s positive rod shape. These colonies were isolated from laying hens (450 isolates in cecum) and broilers (350 isolates in ileum and 1,200 isolates in caecum). Thereafter, these colonies were initial screened for acid and bile salt tolerance in order to eliminate some isolates that did not survive under these conditions. It was found that 200 colonies exhibited well-tolerance in both conditions. The selected 200 colonies of LAB were then identified by sequencing the 16S rRNA gene. The results revealed that 200 LAB isolates belonged to five strains, including *L. acidophilus* (63 colonies), *L. ingluviei* (2 colonies), *L. reuteri* (58 colonies), *L. salivarius* (72 colonies) and *L. saerimneri* (5 colonies).

### Tolerance to acidic pH and bile salt

The tolerance of 200 LAB colonies to acidic pH and bile salt were again tested to select those with the best potential under such conditions. The results indicated that all the strains tested exhibited different survival rates under various acidic pH conditions (Table 2). They showed that *L. acidophilus* and *L. ingluviei* had the highest survival rate, followed by *L. reuteri*, *L. salivarius* and *L. saerimneri* (p<0.05). All *Lactobacillus* strains were able to survive at pH levels of 3.0 and 3.5 for 4 h. While at pH 2.0 and 2.5 the four strains (*L. ingluviei*, *L. reuteri*, *L. acidophilus*, and *L. salivarius*) were able to survive, except for *L. saerimneri* for which the survival rate was retained for only 4.25% and 5.78% after exposure to pH 2.0 and 2.5, respectively for 4 h. Considerable resistance to low pH was observed in strains of *L. acidophilus*, whereas the highest sensitivity to an acidified environment was noted for *L. saerimneri* (p<0.05).

In addition to acidic conditions, the bile salt tolerance of selected *Lactobacillus* strains was also examined for which the results are shown in Table 3. All the isolates tested were able to resist various bile salt concentrations of 0.3% to 1.0%. However, as the bile salt concentration increased, their growth rate decreased while *L. acidophilus*, *L. ingluviei*, and *L. reuteri* exhibited well resistance to bile salt at a concentration of 1.0% (p<0.05).

### Antimicrobial activity

The five isolated strains were tested for their antimicrobial activities against various bacterial pathogens which constitute the main problem in the intestinal tract of poultry. All five strains exhibited antibacterial activity against pathogenic bacteria including *E. coli*, *S. aureus*, *C. jejuni*, *C. perfingens* and *S. enteritidis* under normal conditions (Table 4), in which *L. ingluviei*, *L. acidophilus*, and *L. salivarius* showed strong inhibitory effects. The cell free supernatants of *L. ingluviei*, *L. acidophilus*, and *L. salivarius* exhibited inhibitory activities against all the pathogens. However, *L. saerimneri* did not show any effect on *C. jejuni* and *C. perfringens* and *L. reuteri* did not show any effect on *C. perfringens*. When the supernatant of the five strains was treated with NaOH to achieve a pH of 6.5, it found that the *L. ingluviei*, *L. acidophilus*, and *L. salivarius* strains showed inhibitory activities against all the pathogens. Overall, *L. ingluviei* and *L. acidophilus* demonstrated the most beneficial effects on antimicrobial activity under various conditions.

### Antibiotic susceptibility assay

The sensitivity of Lactobacillus strains to the selected antibiotics is presented in Table 5. This table demonstrates that all the strains tested were interpreted as resistant to ampicillin and erythromycin. On the other hand, all the strains exhibited intermediate susceptibility to tetracycline and sensitivity to chloramphenicol.

### Caco-2 cell adhesion

The adhesion of *Lactobacillus* isolates to intestinal cells was investigated using Caco-2 cells (Table 6). The *L. ingluviei* strain exhibited the strongest adhesion to Caco-2 cells followed by *L. salivarius* and *L. acidophilus*, whereas *L. saerimneri* and *L. reuteri* expressed less strength of adherence.

### Cholesterol removal ability

**Figure 1:**
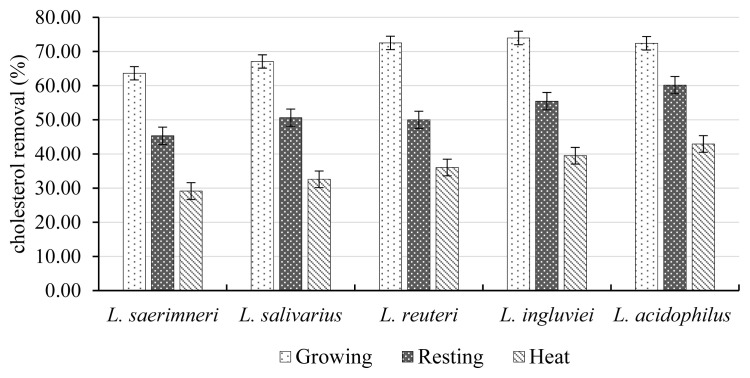
The percentage of cholesterol removal of growing, resting and dead cells of *Lactobacillus* strains isolated from the digestive tract of chickens cultured in De Man, Rogosa and Sharpe supplemented with 100 g/mL water-soluble cholesterol and 0.3% oxgall.

The ability of five isolated bacteria, either growing or non-growing (resting or dead cells) to remove cholesterol was assessed. The removal of cholesterol varied significantly amongst growing, resting and dead cells, ranging from 63% to 72%, 45% to 60%, and 29% to 42%, respectively. *L. reuteri*, *L. ingluviei*, and *L. acidophilus* were more effective in cholesterol removal than the other probiotics (Figure 1).

### Evaluation of probiotics *L. ingluviei* and *L. salivarius* in broiler chickens

#### Cecal microbial populations

The effects of probiotics *L. ingluviei* and *L. salivarius* on the cecal microbial population of broilers are shown in Table 6. It was found that the oral administration of both *L. ingluviei* and *L. salivarius* can increase *Lactobacillus* spp. and *Bifidobacterium* spp. population in cecal content. In addition, these probiotics can also decrease the number of *Enterobacter* and *E. coli* compared to the negative control group (Table 7).

#### SCFA analysis

The effects of *L. ingluviei* and *L. salivarius* on cecal VFA concentrations of broilers are shown in Table 8. These showed that the administration of *L. ingluviei* and *L. salivarius* can increase valeric acid and SCFA concentrations in the cecal content (p<0.05). However, there were no significant differences in the concentrations of acetic acid, propionic acid, butyric acid or branched SCFA.

## DISCUSSION

Probiotics are a potential alternative feed additive to improve the gut health of animals and address issues related to the intensive animal rearing system and the ban on the use of antibiotics as growth promoters. It is well known that LAB is the main probiotic used in animal feed, and that their function is associated with conductive properties for the host health, gut acidification, elimination of unfavorable microflora, improvement of digestive and metabolic processes, stimulation of immunological response, enhancement of intestinal barrier function and maintenance of natural microbial balance [23]. Although several LAB such as *Lactobacillus*, *Lactococcus*, *Bifidobacterium*, *Pediococcus*, *Enterococcus*, and *Propionibacterium* are already established and widely used as probiotics in animal feed [23,24], unfortunately, application in the practical field may vary depending on several factors such as the animal host, diet, hygiene conditions, antibiotic treatment, and stress factors [25]. Therefore, there is still a need to search for new probiotic strains with the greatest potential and benefits for the poultry industry. Probiotics are likely to function efficiently depending on their source and the specificity of their host [24,26]. In the present work, the study of the functional properties of LAB strains from various types of poultry (broilers, slow- growing chickens, and laying hens) were conducted according to acid and bile tolerance, antimicrobial activity, adhesion to epithelial cells and additional characteristics on cholesterol removal. In this study, the 200 LAB isolates belong to five strains, including *L. acidophilus* (63 isolates), *L. ingluviei* (2 isolates), *L. reuteri* (58 isolates), *L. salivarius* (72 isolates), and *L. saerimneri* (5 isolates).

Acid and bile salt tolerances are the most important criteria for selecting strains of probiotic capable of survival in the GIT. The pH of the GIT in chicken varies in different parts; in which retention times and pH in GIT of the chickens have been recorded as follows: crop pH 4.8 and 30 min; proventriculus pH 4.4 and 15 min; gizzard pH 2.6 and 90 min; small intestine pH 6.2 and 90 min; and large intestine pH 6.3 and 15 min [27]. In this study acidic conditions were tested at pH 2.0, 2.5, 3.0, and 3.5 in order to cover the pH values in the gizzard, as well as the tolerance of *Lactobacillus* strains to bile salt (at levels of 0.3% and 1%). It was found that all the five *Lactobacillus* strains tested showed resistance to pH 3.0 at 90 min, but their viability declined to pH 2.0. In particular, *L. acidophilus* and *L. ingluviei* exhibited the strong acid resistance followed by *L. salivarius*. The findings of this study are consistent with those of other previous studies, Ehrmann et al [12] indicated that *Lactobacillus* strains isolated from ducks can survive for 4 h when incubated at pH 2.0 and 3.0, and few of them can even survive for an hour at pH 1.0. In addition, Hutari et al [28] reported that *L. salivarius* and *L. fermentum* isolated from chickens were able to survive at pH 2.5 for 3 h. Our findings also revealed that all *Lactobacillus* strains can resist various bile salt levels (0.3% and 1%), although the survival rate decreased as the bile salt concentration increased (average survival rate of 81% and 72% in bile salt levels of 0.3% and 1%, respectively). These results are similar to those result obtained by Erkkilä and Petäjä [29] with the strains of *Pediococcus acidilactici*, *L. curvatus* and *L. sake* being the most resistant to 0.3% bile salt at pH 6.0. Pennacchia et al [30] reported *Lactobacillus* strains (*L. plantarum* and *L. brevis*) were able to grow in a MRS agar supplemented with 0.3% bile salt. This study indicated that five strains of *Lactobacillus* isolated from the cecum and ileum of chickens have good resistance to acid pH and bile salt, as these properties helped them survive in the GIT of chickens and they adhered to the intestinal cells while exerting beneficial effects.

Probiotics with antibacterial activity against pathogens are a promising alternative to antibiotics [31]. Interestingly, the *Lactobacillus* isolates in this study were highly detectable in cases of *L. salivarius*, *L. ingluviei*, and *L. acidophilus* which showed significant antibacterial activities against all the tested pathogenic bacteria (*E. coli*, *S. aureus*, *C. jejuni*, *C. perfingens*, and *S. enteritidis*). Antagonistic activity by LAB is sustained by the secretion of different antimicrobial substances including SCFAs, bacteriocins, hydrogen peroxide and antimicrobial peptide [31]. Once the pH of the cell free supernatant was neutralized (pH 6.5), all the *Lactobacillus* isolates lost their antagonistic activity against the pathogens tested, with the exception of *L. ingluviei* and *L. acidophilus* which demonstrated weak and moderate antagonistic activity against pathogenic bacteria. In addition, LAB strains from poultry also showed efficacy on antimicrobial activity in the pH range of 1.0 to 4.0, but complete loss of activity at 5.0 to 11.0 pH. The benefit of *Lactobacillus* isolates as shown by our study on antimicrobial activity are likely attributable to the function of organic acid secretion, bacteriocins and other antimicrobial substances [31]. The secretion of bacteriocin by LAB is highly affected by temperature, pH, incubation time and certain other environmental factors. It was also reported that there is optimum secretion of bacteriocin when LAB remains in the pH range of 5.0 and 6.0 [32]. In the present study, all five isolated strains showed antibacterial activity against various bacterial pathogens, including *E. coli*, *S. aureus*, *C. jejuni*, *C. perfingens*, and *S. enteritidis* under normal conditions, which almost possess the problem towards the digestive tract of poultry.

An important requirement of probiotics is that the isolated strain must be safe for animal and human consumption. Antimicrobial resistance is an increasingly serious global threat to human, animal and environmental health. Antibiotic resistance properties in various *Lactobacillus* species appeared to be associated with drug-resistant genes which are mainly located on the chromosome. In the current study, a group of drugs (such as ampicillin, erythromycin, tetracycline, and chloramphenicol), which are commonly used to treat the disease in poultry, have been tested for susceptibility to the five *Lactobacillus* strains. Our study found that all the *Lactobacillus* strains were resistant to a broad range of antibiotics related to various modes of action, such as β-lactam antibiotics (ampicillin) and macrolide antibiotic (erythromycin). In addition, all strains showed intermediate susceptibility and susceptibility to broad-spectrum antibiotics (tetracycline and chloramphenicol). It has been reported that *Lactobacillus* strains can produce β-lactamase which is resistant to β-lactam antibiotics including ampicillin [33]. Dowarah et al [23] also reported high sensitivity to penicillin, ampicillin and chloramphenicol by LAB strains isolated from pigs and poultry. Nevertheless, it has also been documented that *Lactobacillus* are generally susceptible to ampicillin [33]. Jose et al [34] reported that LAB strains isolated from milk, animal rumen and most commercial probiotics exhibited intrinsic resistance to streptomycin, gentamicin and vancomycin, which are aminoglycosides and glycopeptides. The intrinsic antibiotic resistant nature of LAB probiotics suggests their application for both therapeutic and preventive purposes in the treatment and control of intestinal infections, especially when administered concurrently with antibiotics and that GIT microflora recovery can be enhanced by this probiotic.

Adhesion of probiotic strains to the intestinal mucosa is considered as a prerequisite characteristic for potential probiotic microorganisms. As probiotics adhere to the intestinal mucosa, their function can have several beneficial effects on the host gut, such as the prevention of pathogenic colonization, the maintenance of gut mucosal immunity and the healing of damaged mucous membranes [35]. In this study we found that *L. ingluviei* exhibited the strongest adhesion to Caco-2 cells followed by *L. salivarius* and *L. acidophilus*, whereas *L. saerimneri* and *L. reuteri* expressed less strength of adherence. This may indicate that the good adhesiveness of *L. ingluviei* and *L. salivarius* suggest beneficial functions for the health of the host in comparison to other isolated strains. A previous study demonstrated that single or multi-strain LAB probiotics showed excellent adhesion to COLO 205 cells (an epithelial colorectal adenocarcinoma), which could indicate their ability to colonize intestinal epithelial cells and act as a barrier to protect intestinal mucosa from pathogens [36]. Noohi et al [14] reported that *L. brevis* and *L. reuteri* strains showed significant attachment to Caco-2 cells and a high capacity for biofilm formation. In general, LAB adhesion is a complex process initiated from the foremost bacterial contact with the cell membrane of the host enterocytes, followed by various surface interactions. Most LAB can produce cell surface proteins that aid bacteria in binding to intestinal epithelial cells and activate immunoregulation to protect pathogens.

Recently, the incidence of cardiovascular disease in humans has increased, which has a strong correlation with the serum cholesterol level. As a result, much attention has been given to the screening of probiotics that can increase the removal of cholesterol. In this study we found that all five *Lactobacillus* strains have the potential of cholesterol removal either growing or non-growing (resting or dead cells) in which *L. reuteri*, *L. ingluviei*, and *L. acidophilus* were more effective in cholesterol removal than the other strains. This additional function of *Lactobacillus* strains could be useful in applications to improve the quality of production with low meat or egg cholesterol. The function of removing cholesterol is probably due to the bile salt hydrolase in probiotics particularly in the strains *Lactobacillus* and *Bifidobacterium*, in which this enzyme is responsible for the hydrolysis of conjugated bile acids into deconjugated bile acids and amino residues, whose deconjugated forms are less soluble and less absorbed by the intestine, leading to the elimination of excreta [18]. As a result, cholesterol is now used to synthesize new bile acids in a homeostatic response, leading to a reduction in serum cholesterol in animals [18]. The highest cholesterol removal properties of growing cells found in this study indicate that the degree of bound cholesterol may depend on cell growth. It is interesting to note that the resting and dead cells of *Lactobacillus* isolates still maintain a function in cholesterol removal, which is probably due to their cell membrane still having the ability to bind cholesterol. This is in accordance with the report of Lye et al [37] who stated that the membrane bilayer of probiotic cells (*Lactobacillus* and *Bifidobacterium*) have the ability to incorporate cholesterol, especially in the areas of the phospholipid tail, upper phospholipids and polar heads.

In this study, the two strains (*L. ingluviei* and *L. salivarius*) were selected to assess their efficacy in broiler chickens. Since these two probiotic strains have shown the greatest advantages in the *in vitro* test, although previous studies still lack information about broilers. Probiotics *L. ingluviei* and *L. salivarius* were found to increase the cecal population of *Lactobacillus* and B*ifidobacterium* while reducing *Enterobacteria* and *E. coli* relative to the control group. This is consistent with the results of the *in vitro* tests in the present study, which showed that the *L. ingluviei* and *L. salivarius* were against all bacterial pathogens tested (*E. coli*, *S. aureus*, *C. jejuni*, *C. perfingen*, and *S. enteritidis*). Angelakis et al [38] reported that in mice inoculated with *L. ingluviei*, the number of *Lactobacillus* and Firmicutes in the feces increased significantly. This is in accordance with the findings of Shokryazdan et al [39] who reported that supplementation of the three *L. salivarius* strains at levels of 0.5 or 1 g/kg diet can increase the populations of beneficial bacteria such as *Lactobacillus* and *Bifidobacterium* and decrease harmful bacteria such as *E. coli* and total aerobes. Sureshkumar et al [40] also reported that the oral administration of *L. salivarius* can increase the population of beneficial bacteria and reduce pathogenic bacteria in the fecal microbiota.

*L. ingluviei* and *L. salivarius* were observed to improve the production of valeric acid and total SCFAs. In general, SCFAs are metabolites of bacteria in the gut of which the concentration may vary depending on the prevailing microbiota, the type of fermentation substrate and the period of fermentation. In this study, a significant increase in valeric acid and total SCFAs in cecal digesta may be associated with an increase in the population of *Lactobacillus* and *Bifidobacterium*, which were more abundant in chicken groups administered with *L. ingluviei* and *L. salivarius*. SCFAs have been reported to decrease cecal pH and indirectly inhibit pathogenic microorganisms susceptible to pH changes, as well as passing into the cells of pathogens causing a change of positive and negative ions resulting in cells becoming unbalanced and inhibiting the growth of pathogens [41]. In addition, Tsukagoshi et al [42] reported that *L. ingluviei* C37 exerted anti-inflammatory effects by modulating cytokine profiles in a mice model. It is interesting to note that the *L. ingluviei* and *L. salivarius* increased the concentration of valeric acid approximately three times more than the control group. The valeric acid is mostly produced by certain members of gut microbiota belonging to Firmicutes bacteria [43]. In addition, valeric acid was identified as a potential therapeutic target for a variety of disease pathologies. The findings of Onrust et al [44] revealed supplementation of valeric acid glyceride esters can improve feed efficiency, gut morphology and the density of glucagon-like peptide-2-producing enteroendocrine cells and reduce the incidence of necrotic enteritis. This suggests that *L. ingluviei* and *L. salivarious* could be beneficial in improving gut health and preventing disease. Future studies will be necessary to investigate their efficacy on the growth performance of broilers.

## CONCLUSION

In this study, the *Lactobacillus* strains (*L. ingluviei*, *L. acidophilus* and *L. salivarius*) isolated from the gut contents of chickens exhibited a strong resistance to acid and bile salt, antibacterial activity, antibiotic tolerance and high adherence to intestinal epithelial cell. The efficacy of both selected probiotics *L. ingluviei* and *L. salivarius* in broiler chickens was found to improve gut health by increasing the population of *Lactobacillus* and *Bifidobacterium* with an associated increase in valeric acid and total SCFA. The results indicate that all five *Lactobacillus* strains, especially *L. ingluviei* and *L. salivarius* have probiotic properties. Future studies should assess their potential for antibiotic replacement and improvement of broiler growth performance.

## Figures and Tables

**Table 1:** Nutrient composition of the experimental diets (as-fed basis)

Items	Starter diets (1 to 10 d)	Grower diets (11 to 14 d)
Ingredients (%)		
Corn	53	55.3
Soybean meal, 44% crude protein	32.56	31
Full-fat soybean, 36% crude protein	6.8	5
Cassava starch	0.3	0.3
Rice bran oil	3	4.06
Calcium carbonate	1.45	1.25
Monocalcium phosphate	1.4	1.44
Sodium chloride	0.51	0.5
Premix^1)^	0.5	0.5
L-lysine	0.14	0.23
DL-methionine	0.29	0.3
L-threonine	0.05	0.12
Calculated composition (%)		
Metabolizable energy (kcal/kg)	3,008	3,086
Calcium	0.92	0.84
Available phosphorus	0.42	0.42
Digestible lysine	1.17	1.17
Digestible methionine	0.57	0.57
Digestible methionine + cystine	0.87	0.86
Digestible threonine	0.76	0.79
Analyzed composition (%)		
Dry matter	90.61	90.67
Crude protein	21.12	20.09
Crude fat	6.32	7.12

1) Premix (0.5%) provided the following per kilogram of diet: vitamin A, 15,000 IU; vitamin D_3_, 3,000 IU; vitamin E, 25 IU; vitamin K_3_, 5 mg; vitamin B_1_, 2 mg; vitamin B_2_, 7 mg; vitamin B_6_, 4 mg; vitamin B_12_, 25 μg; pantothenic acid, 11.04 mg; nicotinic acid, 35 mg; folic acid, 1 mg; biotin, 15 μg; choline chloride, 250 mg; Cu, 1.6 mg; Mn, 60 mg; Zn, 45 mg; Fe, 80 mg; I, 0.4 mg; Se, 0.15 mg.

**Table 2:** Acid tolerance of *Lactobacillus* strains isolated from the digestive tract of chickens in different pH

Strain	Survival rate (%)			

	pH 2.0	pH 2.5	pH 3.0	pH 3.5
*L. salivarius*	18.00^c^	16.04^c^	21.20^c^	39.20^c^
*L. reuteri*	22.56^c^	33.75^b^	57.14^b^	67.70^b^
*L. acidophilus*	45.50^a^	59.00^a^	78.40^a^	89.45^a^
*L. ingluviei*	34.00^b^	45.70^a^	68.50^a^	78.54^ab^
*L. saerimneri*	4.25^d^	5.78^c^	27.09^c^	30.45^c^
Pooled SEM	2.91	3.11	2.85	9.17
p-value	0.023	0.024	<0.001	0.032

Pooled SEM, standard error of the means.

a–d Means with different superscripts in a column are significantly different (p<0.05).

**Table 3:** The bile salt tolerance of *Lactobacillus* strains isolated from the digestive tract of chickens in different bile salt

Strain	Survival rate (%)	

	0.30% bile salt	1.00% bile salt
*L. salivarius*	70.00^b^	59.45^b^
*L. reuteri*	89.45^a^	79.55^a^
*L. acidophilus*	93.34^a^	88.56^a^
*L. ingluviei*	84.46^a^	78.44^a^
*L. saerimneri*	69.54^b^	55.43^b^
Pooled SEM	8.75	6.33
p-value	0.024	<0.001

Pooled SEM, standard error of the means.

a,b Means with different superscripts in a column are significantly different (p<0.05).

**Table 4:** Inhibitory effects of *Lactobacillus* strains isolated from the digestive tract of chickens against pathogenic bacteria^1)^

Treatment	Strain	Pathogenic bactheria^2)^				

		*S. aureus*	*C. jejuni*	*E. coli*	*C. perfringens*	*Salmonella* spp.
Normal cell	*L. saerimneri*	+++	+	+++	+	+++
	*L. salivarius*	+++	+++	+++	++	+++
	*L. reuteri*	+++	++	+++	+	+++
	*L. ingluviei*	+++	+++	+++	+++	+++
	*L. acidophilus*	+++	+++	+++	+++	+++
Cell free supernatant	*L. saerimneri*	++	-	++	-	+
	*L. salivarius*	+++	+	++	+	+
	*L. reuteri*	++	+	++	-	+
	*L. ingluviei*	+++	+	+++	++	+++
	*L. acidophilus*	+++	++	+++	++	++
Cell free supernatant neutralized^3)^	*L. saerimneri*	-	-	+	-	+
	*L. salivarius*	+	+	+	+	+
	*L. reuteri*	+	-	+	-	-
	*L. ingluviei*	+	+	+	+	++
	*L. acidophilus*	++	+	+	+	++

1) Inhibition zone (mm): no inhibition (-); weak (+) <14); ++, good (15 to 19); +++, strong (>20).

2) The pathogenic groups that almost possess the problem towards the intestinal tract of poultry.

3) Supernatant treated with NaOH to obtain a pH 6.5.

**Table 5:** Antibiotic susceptibility of *Lactobacillus* strains isolated from the digestive tract of chickens

Strain	Ampicillin	Erythromycin	Tetracycline	Chloramphenicol
*L. saerimneri*	R	R	I	S
*L. salivarius*	R	R	I	S
*L. reuteri*	R	R	I	S
*L. ingluviei*	R	R	I	S
*L. acidophilus*	R	R	I	S

S, sensitive; I, intermediate and R, resistant.

**Table 6:** Adhesion ability of the *Lactobacillus* strains isolated from the digestive tract of chickens to Caco-2 cell

Strain	Adhesion capacity (%)
*L. salivarius*	47.77^b^
*L. reuteri*	36.00^c^
*L. acidophilus*	48.78^b^
*L. ingluviei*	56.80^a^
*L. saerimneri*	34.55^c^
Pooled SEM	1.02
p-value	0.035

Pooled SEM, standard error of the means.

a–c Means with different superscripts in a column are significantly different (p<0.05).

**Table 7:** Effects of probiotics *L. ingluviei* and *L. salivarius* administration on cecal microbial population of broiler chickens at 14 days of age (log10 of copy number/g DNA extract)

Item	Control	*L.ingluviei*	*L.salivarius*	Pooled SEM	p-value
*Lactobacillus*	7.80^b^	9.86^a^	9.97^a^	0.11	0.016
*Bifidobacterium*	6.70^b^	8.86^a^	8.87^a^	0.13	0.017
*Enterobacter*	8.79^a^	5.78^c^	7.10^b^	0.25	0.029
*E. coli*	8.25^a^	7.44^b^	6.66^c^	0.17	0.013

Pooled SEM, standard error of the means.

a–c Means with different superscripts in a row are significantly different (p<0.05).

**Table 8:** Effects of probiotics *L. ingluviei* and *L. salivarius* administration on cecal SCFA concentrations (umol/g of digesta) of broiler chickens at 14 days of age

Item	Control	*L. inguluviei*	*L. sarivarius*	Pooled SEM	p-value
Acetic acid	30.88	39.11	38.83	3.51	0.569
Propionic acid	4.99	5.46	3.20	0.50	0.216
Butyric acid	4.39	6.73	4.35	0.73	0.346
Valeric acid	40.31^b^	116.58^a^	107.37^a^	11.52	<0.001
Branched SCFA^1)^	0.52	0.94	0.66	0.21	0.728
Total SCFA	81.08^b^	168.82^a^	154.40^a^	13.39	<0.001

SCFA, short chain fatty acid; Pooled SEM, standard error of the means.

1) Branched SCFA = isobutyric acid + isovaleric acid.

a,b Means with different superscripts in a row are significantly different (p<0.05).
